# Disrupting the interaction between AMBRA1 and DLC1 prevents apoptosis while enhancing autophagy and mitophagy

**DOI:** 10.1242/bio.060380

**Published:** 2024-11-26

**Authors:** Kate Hawkins, Meg Watt, Sébastien Gillotin, Maya Hanspal, Martin Helley, Jill Richardson, Nicola Corbett, Janet Brownlees

**Affiliations:** MSD R&D Innovation Centre, 120 Moorgate, London, EC2M 6UR

**Keywords:** Autophagy, Mitophagy, Apoptosis, Neurodegeneration

## Abstract

AMBRA1 has critical roles in autophagy, mitophagy, cell cycle regulation, neurogenesis and apoptosis. Dysregulation of these processes are hallmarks of various neurodegenerative diseases and therefore AMBRA1 represents a potential therapeutic target. The flexibility of its intrinsically disordered regions allows AMBRA1 to undergo conformational changes and thus to perform its function as an adaptor protein for various different complexes. Understanding the relevance of these multiple protein–protein interactions will allow us to gain information about which to target pharmacologically. To compare potential AMBRA1 activation strategies, we have designed and validated several previously described mutant constructs in addition to characterising their effects on proliferation, apoptosis, autophagy and mitophagy in SHSY5Y cells. AMBRA1^TAT^, which is a mutant form of AMBRA1 that cannot interact with DLC1 at the microtubules, produced the most promising results. Overexpression of this mutant protected cells against apoptosis and induced autophagy/mitophagy in SHSY5Y cells in addition to enhancing the switch from quiescence to proliferation in mouse neural stem cells. Future studies should focus on designing compounds that inhibit the protein–protein interaction between AMBRA1/DLC1 and thus have potential to be used as a drug strategy for neurodegeneration.

## Teaser

**Summary:** This paper demonstrates that blocking the interaction between DLC1 and AMBRA1 shifts the balance away from apoptosis and towards autophagy/mitophagy, thus representing a potential therapeutic strategy for neurodegeneration.

## INTRODUCTION

Activating molecule in Beclin1-regulated autophagy (AMBRA1) is an intrinsically disordered protein (IDP) with a molecular mass of approximately 130 kDa. IDPs participate in many biological processes, and the flexibility of their disordered regions allows for conformational changes and thus binding to various different proteins depending on the cellular context. AMBRA1 acts as a scaffolding protein in this way to form complexes to facilitate proliferation, apoptosis, autophagy and mitophagy (Cianfanelli et al., 2015; Di Leo et al., 2021; Di Rita et al., 2018b; Chaikovsky et al., 2021). Interestingly, these processes (particularly autophagy) have been implicated in neurodevelopment and adult neurogenesis (Gillotin et al., 2021). Mutations in AMBRA1 cause neural tube defects in both mice (Fimia et al., 2007) and humans (Ye et al., 2020) and downregulation of AMBRA1 has been shown to lead to reduced survival of neural stem cells (NSCs) (Yazdankhah et al., 2014; Vazquez et al., 2012). In addition, one group observed enhanced reactivation of quiescent NSCs in BECLIN1/BCL2 mutant mouse NSCs in which BECLIN1 is constitutively active (Wang et al., 2022). However, this is yet to be shown for AMBRA1 specifically.

The main role of AMBRA1 is in the autophagy signalling network, which mediates lysosomal degradation of damaged or redundant cellular components (Aman et al., 2021). When autophagy is initiated, for example by nutrient depletion, mTOR is inactivated thus allowing ULK1 activation. ULK1 then phosphorylates BECLIN1 and AMBRA1 to promote AMBRA1 release from the microtubules where it is tethered via dynein light chain (DLC)1, and subsequent activation of the VPS34 complex. The BECLIN1/VPS34 complex is then directed to the endoplasmic reticulum (ER) where it participates in autophagosome formation from the ER membrane (Fimia et al., 2011; Tang et al., 2016). In addition, a positive feedback mechanism occurs whereby AMBRA1 stabilises ULK1 by assisting TRAF6 to carry out K63 chain ubiquitination of ULK1, resulting in its self-association. Upon autophagy termination, other E3 ligases such as CULLIN4 and RNF2 (Xia et al., 2014) interact with AMBRA1 to mediate its degradation. For instance, CULLIN4 in association with DDB1 binds to AMBRA1 via its WD40 domain (Antonioli et al., 2014).

AMBRA1 also plays a key role in both PARKIN dependent and independent mitophagy. In the former process, AMBRA1 associates with the E3 ligase PARKIN to mediate ubiquitination and subsequent degradation of outer mitochondrial membrane proteins and in the latter the HUWE1 E3 ligase performs this function in the place of PARKIN. It has been shown that AMBRA1 directly binds to LC3 via its LIR domain (Strappazzon et al., 2015) and that, during PARKIN independent mitophagy, IKKα induces phosphorylation of S1014, which stabilises the interaction between AMBRA1 and LC3 (Di Rita et al., 2018b).

Currently, there is no drug that has been described to activate AMBRA1. However, tagging a mitochondrial localisation sequence onto the AMBRA1 protein (AMBRA1^ACTA^) has been shown not only to induce mitophagy but to counteract the oxidative stress and apoptosis induced by either rotenone or 6-OHDA in SHSY5Y cells (Di Rita et al., 2018a). This represents an interesting mechanism that could mimic some therapeutic strategies currently developed for neurodegeneration.

We have designed and overexpressed in SHSY5Y cells (which otherwise express negligible levels of AMBRA1 protein in our hands) various mutant AMBRA1 constructs representing potential activation strategies (Table S1). Following validation of these constructs we have qualitatively characterised their effects on proliferation, apoptosis, autophagy and mitophagy. AMBRA1^TAT^, which cannot bind to dynein light chain (DLC)1 at the microtubules, was shown to be the most promising mutant, inducing a clear switch away from apoptosis and towards autophagy and mitophagy. We then went on to show that this mutant construct could enhance the transition from quiescence to proliferation in mouse neural stem cells (mNSCs) as a model of neurogenesis. Together, these findings suggest that devising pharmacological strategies to target the AMBRA1/DLC1 interaction may be a promising strategy to tackle known impaired signalling pathways in neurodegeneration.

## RESULTS

### AMBRA1^TAT^ expression protects cells against apoptosis and induces autophagy and mitophagy

**Fig. 1.**
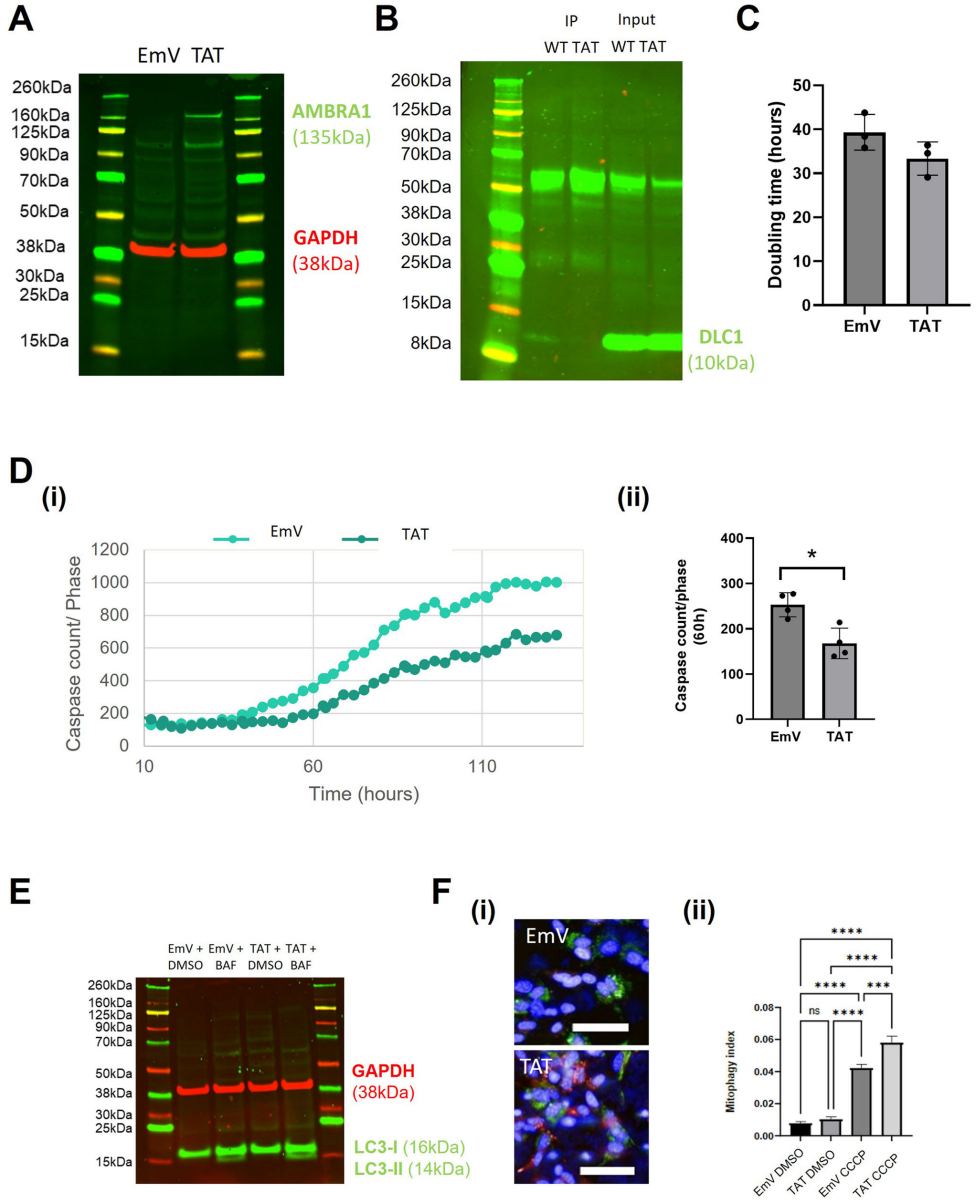
**AMBRA1^TAT^ SHSY5Y validation and characterisation.** (A) Western blot of AMBRA1^TAT^ and EmV (control) SHSY5Y cells to show AMBRA1 expression. (B) Western blot of DLC1 protein expression in Co-IP samples of AMBRA1^TAT^ and AMBRA1^WT^ SHSY5Y cells. (C) AMBRA1^TAT^ and EmV SHSY5Y doubling time measured in the Incucyte over a period of multiple days. (D) (i) AMBRA1^TAT^ and EmV SHSY5Ys stained with Caspase-3/7 Green and NucRed and imaged in the Incucyte for 130 h in growth media. Caspase levels were normalised to the phase area of each image. (ii) Sample mean±s.d. (E) Western blot analysis of LC3-II expression in AMBRA1^TAT^ and EmV SHSY5Y cells treated with bafilomycin in growth media for 4 h. (F) Normalized quantification of the mitophagy index of AMBRA1^TAT^ and EmV SHSY5Y cells transduced with mtKeima lentivirus and treated with 1uM CCCP. Scale bar: 50 µm. Sample mean±s.d.***P<0.0005, ****P<0.0001 (two-tailed unpaired *t*-test). All data presented is representative of three experimental repeats. *N*=3.

AMBRA1^TAT^ is a mutant form of AMBRA1 that cannot interact with DLC1, meaning that AMBRA1 can translocate to the ER and initiate autophagosome formation. Firstly, we confirmed the lentiviral overexpression of AMBRA1^TAT^ in the SHSY5Y cells by western blotting. This showed increased levels of AMBRA1 expression in the AMBRA1^TAT^ mutant compared to the empty vector (EmV) control (Fig. 1A). Next, we carried out Co-immunoprecipitation (CoIP) to assess the interaction between AMBRA1 and DLC1. As expected, the levels of AMBRA1/DLC1 binding were decreased in the AMBRA1^TAT^ mutant cells compared to AMBRA1^WT^ overexpressing cells (Fig. 1B).

When the validation of the cell model was complete, we then characterised the AMBRA1^TAT^ SHSY5Y cells. We saw no change in proliferation between the AMBRA1^TAT^ cells and the EmV control cells (Fig. 1C), however expression of the TAT mutant construct was able to protect against apoptosis (Fig. 1D) in addition to increasing the levels of LC3 lipidation in the cells, which is indicative of basal autophagy levels, ∼1.43 fold±0.06 (Fig. 1E, Table S3). AMBRA1^TAT^ also significantly increased the mitophagy index in the presence of CCCP, which is indicative of the levels of mitophagy in the cells (Fig. 1F). Taken together, these results suggest that blocking the AMBRA1/DLC1 interaction may induce a phenotype that would be beneficial in a neurodegeneration context.

### AMBRA1^WD40^ expression decreases autophagy flux

**Fig. 2.**
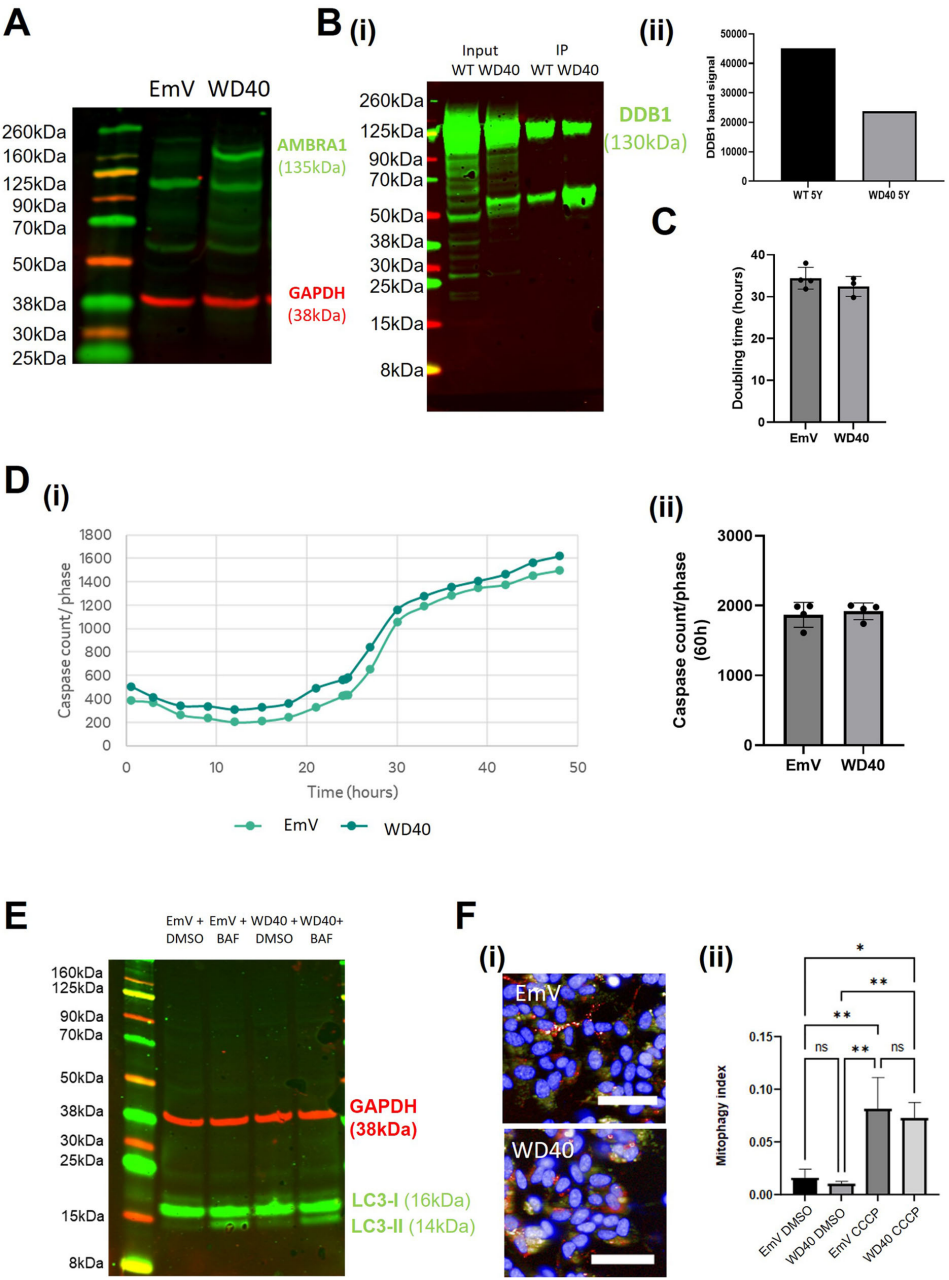
**AMBRA1^WD40^ SHSY5Y validation and characterisation.** (A) Western blot of AMBRA1^WD40^ and EmV SHSY5Y cells to show AMBRA1 expression. (B) (i-ii) Western blot of DDB1 protein in Co-IP samples of AMBRA1^WD40^ and AMBRA1^WT^ SHSY5Y cells. (C) AMBRA1^WD40^ and EmV SHSY5Y cells doubling time measured in the Incucyte over a period of multiple days. (D) (i) AMBRA1^WD40^ and EmV SHSY5Y cells stained with Caspase-3/7 Green and NucRed and imaged in the Incucyte for approximately 50 h in growth media. Caspase levels were normalised to the phase area of each image. (ii) Sample mean±s.d. (E) Western blot analysis of LC3-II expression in AMBRA1^WD40^ and EmV SHSY5Y cells treated with bafilomycin in growth media for 4 h. (F) Normalized quantification of the mitophagy index of AMBRA1^WD40^ and EmV SH-SY5Y cells transduced with mtKeima virus and treated with 1uM CCCP. Sample mean±s.d. *P<0.05, **P<0.01, scale bar: 50 µm. All data presented is representative of three experimental repeats. *N*=3.

AMBRA1^WD40^ has a mutation in the WD40 domain that interacts with DDB1, inhibiting AMBRA1 ubiquitination and its subsequent degradation. There should therefore be more unbound AMBRA1 in the cell to form autophagy and mitophagy-related complexes. Western blotting confirmed that AMBRA1^WD40^ overexpressing SHSY5Y cells had increased levels of AMBRA1 expression compared to the control cells (Fig. 2A). In addition, we confirmed that the interaction between AMBRA1 and DDB1 was reduced ∼0.5 fold in the AMBRA1^WD40^ overexpressing cells compared to the AMBRA1^WT^ overexpressing cells using CoIP (Fig. 2B). The AMBRA1^WD40^ overexpressing cells did not show altered proliferation, apoptosis or mitophagy (Fig. 2C-D,F). However, AMBRA1^WD40^ overexpression decreased the levels of LC3 lipidation in the cells ∼0.62 fold±0.09 (Fig. 2E; Table S3). Together, these results suggest that targeting the WD40 domain does not produce a phenotype that would be beneficial in a neurodegeneration context.

### AMBRA^S1014^ expression increases autophagy flux

**Fig. 3.**
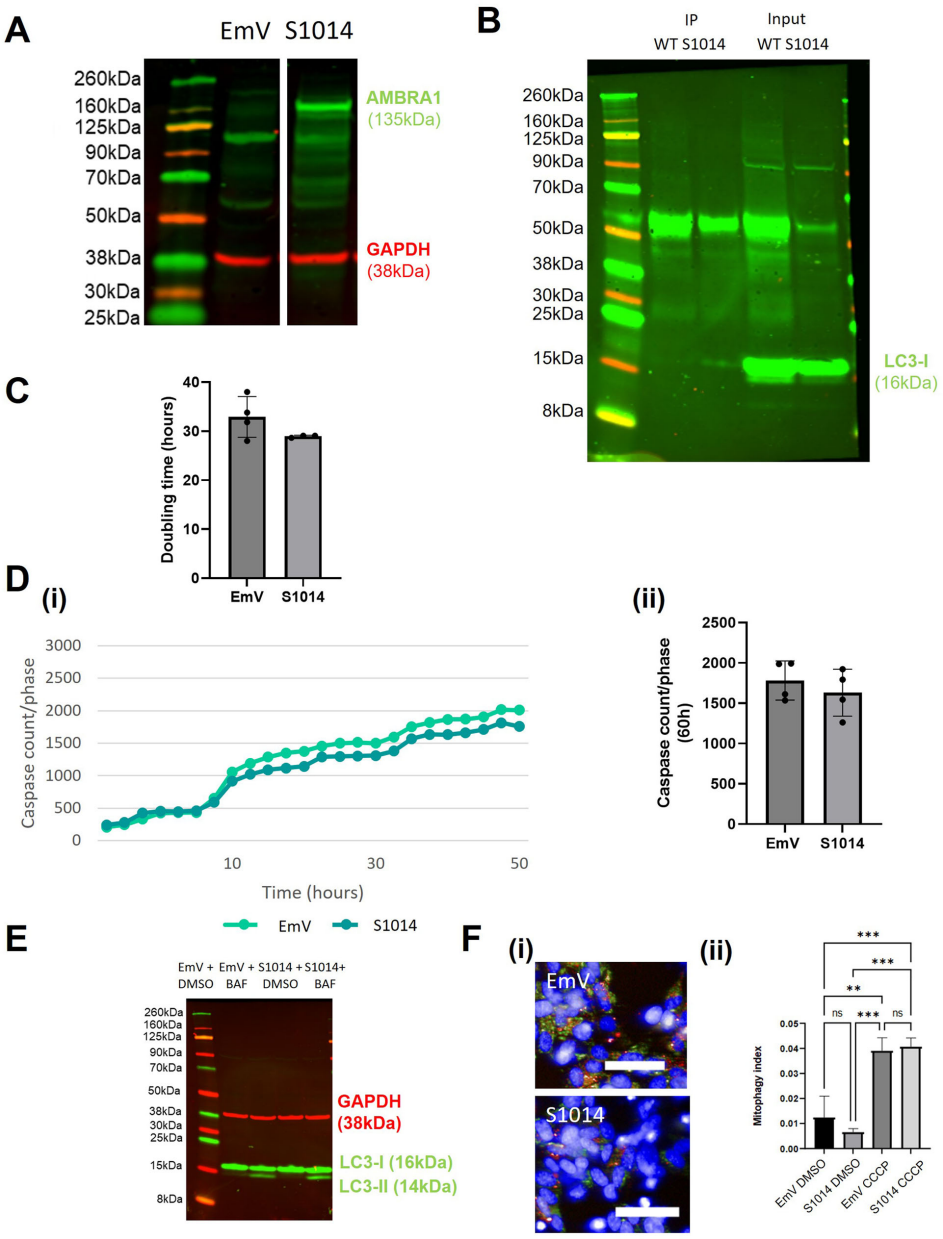
**AMBRA1^S1014^ SHSY5Y validation and characterisation.** (A) Western blot of AMBRA1^S1014^ and EmV SHSY5Y cells to show AMBRA1 expression. Note that this is from the same blot as Fig. 2A. (B) Western blot of LC3 protein in Co-IP samples of AMBRA1^S1014^ and AMBRA1^WT^ SHSY5Y cells. (C) AMBRA1^S1014^ and EmV SHSY5Y cells doubling time measured in the Incucyte over a period of multiple days. (D) (i) AMBRA1^S1014^ and EmV SHSY5Y cells stained with Caspase-3/7 Green and NucRed and imaged in the Incucyte for approximately 70 h in growth media. Caspase levels were normalised to the phase area of each image. (ii) Sample mean±s.d. (E) Western blot analysis of LC3-II expression in AMBRA1^S1014^ and EmV SHSY5Y cells treated with growth media and bafilomycin for 4 h. (F) Normalized quantification of the mitophagy index of AMBRA1^S1014^ and EmV SH-SY5Y cells transduced with mtKeima virus. Sample mean±s.d.; **P<0.01, ***P<0.0005. All data presented are representative of three experimental repeats. *N*=3.

IKKα has been shown to phosphorylate the S1014 residue of AMBRA1, thus stabilising its interaction with LC3 (Di Rita et al., 2018b), therefore we generated a S1014 phospho-mimetic mutant, AMBRA1^S1014^. As expected, there were increased levels of AMBRA1 expression in the AMBRA1^S1014^ mutant cells compared to the EmV cells (Fig. 3A). To determine whether the AMBRA1/LC3 interaction was increased, we carried out CoIP, which confirmed that the AMBRA1^S1014^ overexpressing cells had increased levels of interaction between LC3 and AMBRA1 compared to AMBRA1^WT^ overexpressing cells (Fig. 3B). Expression of AMBRA1^S1014^ did not affect proliferation, apoptosis or mitophagy (Fig. 3C-D,F) but did increase the levels of LC3 lipidation ∼1.31 fold±0.1 (Fig. 3E, Table S3). Therefore, the only mutant that was able to affect apoptosis, autophagy and mitophagy in beneficial ways was the TAT mutant.

### AMBRA1^TAT^ expression enhances the reactivation of quiescent mNSCs

**Fig. 4.**
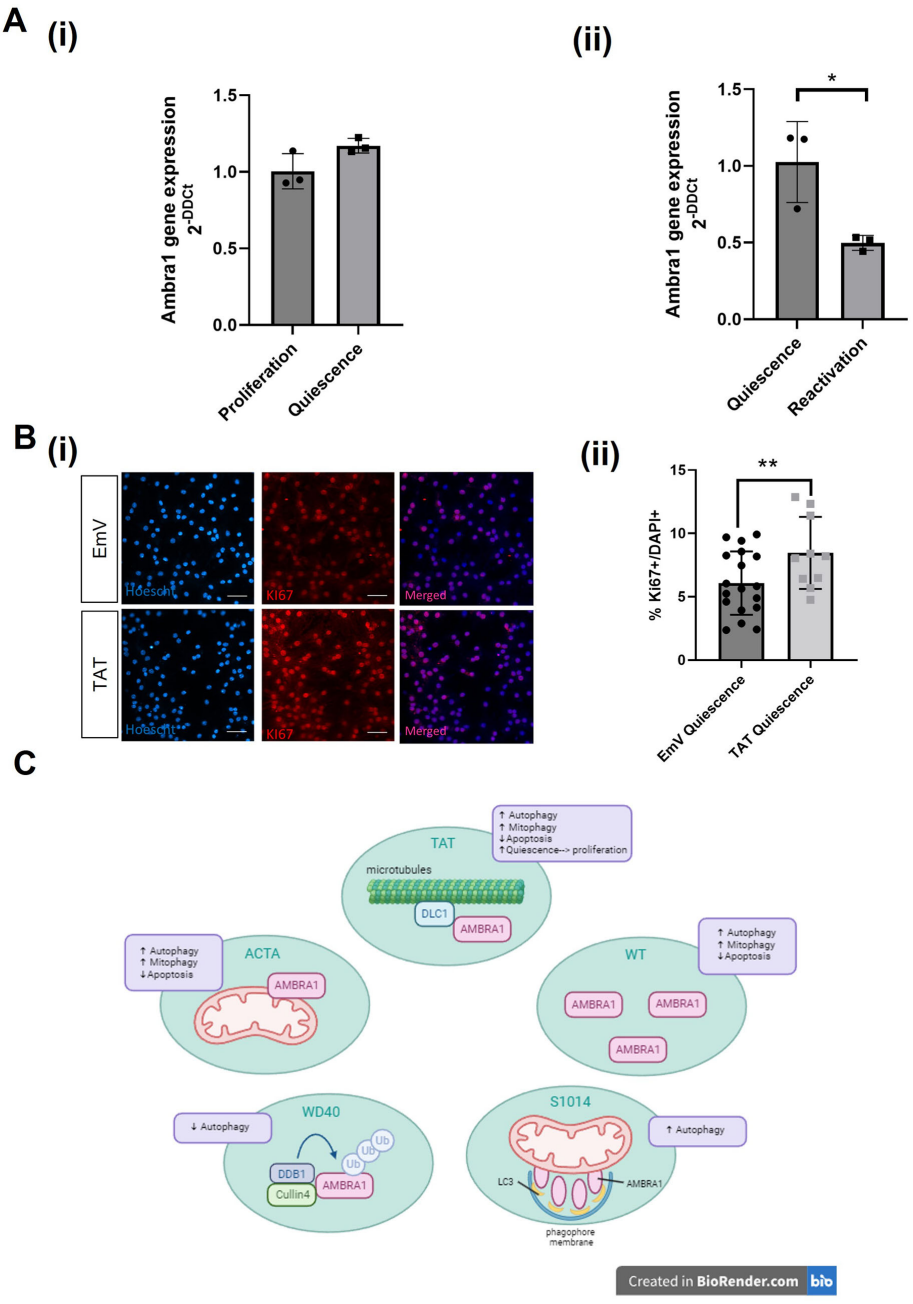
**AMBRA1^TAT^ overexpression in mouse neural stem cells.** (A) Quantification of the fold change of Ambra1 transcript expression in mNSCs that have been (i) induced to become quiescent and (ii) reactivated from quiescence to proliferation. Two-tailed unpaired *t*-test where *P<0.05. (Bi-ii) Immunostaining of Hoescht (blue) and Ki-67 (red) in proliferating and quiescent mNSCs. Quiescence was induced using BMP-4. Scale bar: 50 µm. Images taken at 40× magnification. Averages taken from eight fields of view. Error bars±s.e.m. Two-tailed unpaired *t*-tests where **P<0.01. *N*=3. (C) Summary of findings. Created with Biorender.com. All data presented is representative of three experimental repeats. *N*=3.

AMBRA1 has been implicated in neurodevelopment and recent studies have also implicated autophagy as a key process during adult neurogenesis. Indeed, regulation of autophagy has emerged as a key signalling pathway to modulate the pool of NSCs and dysregulation of autophagy is thought to be one mechanism preventing quiescent cells from reactivating into a proliferative state causing cognitive impairment in several neurodegenerative diseases (Gillotin et al., 2021). We therefore investigated whether AMBRA1 affects the shift from quiescence to proliferation in a well-established *in vitro* model (Martynoga et al., 2013). First, we assessed the levels of *Ambra1* expression in mNSCs undergoing the switch from proliferation to quiescence. *Ambra1* expression showed a trend towards increasing during this transition (Fig. 4Ai) whereas there was a significant decrease in *Ambra1* expression levels 24 h after reactivation from quiescence (Fig. 4Aii). Since activation of autophagy has been proposed as one possible mechanism to promote reactivation of quiescent NSC *in vitro* and *in vivo* (Leeman et al., 2018), we next used the AMBRA1^TAT^ mutant, due to its gain-of-function related to autophagy, to assess whether activation of AMBRA1 could enhance the cellular transition from quiescence to proliferation. Interestingly, overexpression of AMBRA1^TAT^ in mNSCs significantly increased the proportion of Ki-67^+^ proliferative cells compared to the EmV control (Fig. 4Bi-ii). These results suggest that activation of AMBRA1 by disruption of the AMBRA1/DLC1 interaction may help promote the reactivation of quiescent NSCs, a major roadblock to sustain adult neurogenesis in neurodegenerative diseases.

### AMBRA1^ACTA^ expression induces mitophagy and protects cells against apoptosis

AMBRA1^ACTA^ is a form of AMBRA1 with a mitochondrial localisation sequence which should induce translocation of AMBRA1 to the outer mitochondrial membrane (OMM) to induce mitophagy. In order to validate that AMBRA1^ACTA^ translocates to the mitochondria we performed immunofluorescent staining of AMBRA1 and TOM20, an OMM marker, which demonstrated that these two proteins were both localised to the mitochondria in the AMBRA1^ACTA^ but not the control cells (Fig. S1A). AMBRA1^ACTA^ overexpressing cells also exhibited increased AMBRA1 protein expression (Fig. S1B) in addition to an unchanged proliferation rate (Fig. S1C), protection against apoptosis (Fig. S1D), increased levels of LC3 lipidation ∼1.27 fold±0.04 (Fig. S1E; Table S3) and significantly increased mitophagy (Fig. S1F). In conclusion, this mutant has desirable effects on apoptosis and autophagy/mitophagy but does not represent a viable drug strategy.

## DISCUSSION

AMBRA1 is a scaffold protein that performs as a platform for various different molecular processes. In particular, it acts at the crossroads between autophagy and apoptosis (Fimia et al., 2013). Furthermore, processes that are influenced by AMBRA1 have been implicated in neurodevelopment and neurogenesis (Vazquez et al., 2012; Yazdankhah et al., 2014; Wang et al., 2022), making AMBRA1 activation a potential therapeutic strategy for neurodegenerative disease. However, a chemical strategy for AMBRA1 activation is yet to be established.

Di Rita et al., (2018b) previously showed that AMBRA1 localisation to the OMM via the overexpression of AMBRA1^ACTA^ in HeLa cells can stimulate mitophagy. Our findings corroborated this, demonstrating significantly increased levels of both autophagy and mitophagy in SHSY5Y cells overexpressing this construct. Interestingly, overexpression of this mutant construct also protected the cells against apoptosis, which could be due to the increased mitophagy decreasing the levels of damaged mitochondria and thus improving the overall health of the cells.

The AMBRA1^WD40^ mutant construct was designed to prevent the interaction between AMBRA1 and DDB1. Since the DDB1/Cullin4 complex ubiquitinates AMBRA1 this mutant should have lower levels of AMBRA1 ubiquitination and subsequent degradation, similar to what was observed by Antonioli et al. (2014). We hypothesised that the AMBRA1 accumulation that results would allow the formation of complexes involved in autophagy and mitophagy and thus stimulate these processes. However, autophagic flux was found to be lower in the AMBRA1^WD40^ mutant cells and there was no difference in any of the other parameters compared to the control. These findings were unexpected and suggest that alternative complexes, such as the RNF2/WASH complex, that initiates proteasomal degradation (Xia et al., 2014) of AMBRA1, do this to a greater extent in the absence of the interaction between AMBRA1 and DDB1/CULLIN4. The WD40 domain of AMBRA1 is the only domain with a known structure and would therefore be the most straightforward to target pharmacologically. Despite this, our results would not support this as a viable drug strategy for neurodegeneration since AMBRA1/DDB1 protein–protein interaction inhibitor (PPI) compounds would likely block autophagy rather than promote it.

Overexpression of the AMBRA1^S1014^ mutant construct in SHSY5Y cells affected LC3 lipidation but none of the other parameters tested. These findings were expected in that increasing the interaction between AMBRA1 and LC3 should increase autophagy, but you might also expect this to increase the levels of mitophagy since the AMBRA1/LC3 interaction is also involved in this process. Indeed, Di Rita et al., (2018b) observed increased mitophagy in this phospho-mimetic mutant. However, this was in HeLa cells, which do not have an active PARKIN-dependent mitophagy pathway, and therefore PARKIN-independent mitophagy such as that coordinated by AMBRA1/HUWE1 is likely to play a more predominant role. We used SHSY5Y cells in preference to HeLa cells due to the differences in the levels of AMBRA1 expression between the two cell lines. HeLa cells have high levels of AMBRA1 protein expression compared to SHSY5Y cells (data not shown) in which the levels are extremely low and therefore endogenous AMBRA1 is not likely to interfere with the mutant AMBRA1 constructs that we overexpressed as it would in HeLa cells.

Of all the mutant constructs characterised, AMBRA1^TAT^ showed the most promise in that its overexpression significantly increased both autophagy and mitophagy parameters whilst simultaneously protecting cells from apoptosis. In addition, there was no effect on cellular proliferation, which ensures the tumorigenesis risk of this activation strategy is low. AMBRA1^ACTA^ and AMBRA1^WT^ overexpression (Figs S1 and S2) resulted in the same phenotype however these approaches do not represent viable pharmacological strategies. It has previously been shown that AMBRA1^TAT^, which exhibits reduced binding to DLC1, increases the levels of autophagy in cells (Di Bartolomeo et al., 2010). However, we show for the first time that this mutant can also induce mitophagy. This is unsurprising since the molecular mechanisms are highly conserved between the two processes.

Moreover, we show here that mNSCs transduced with the AMBRA1^TAT^ mutant construct exhibit enhanced reactivation from quiescence to proliferation. This switch is central to adult neurogenesis as most NSCs remain in quiescence and their inability to switch back to a proliferative state increases with age and in several neurodegenerative diseases (Gillotin et al., 2021). In this context, AMBRA1^TAT^ is likely increasing autophagy, similar to what we observed in the SHSY5Y cells, which could supply degradable substrates to the lysosomes to activate quiescent NSCs supporting a proposed process to enhance adult neurogenesis (Leeman et al., 2018).

Since the AMBRA1^TAT^ mutant represents a potential PPI strategy between AMBRA1/DLC1, we suggest that this approach in particular should be pursued if AMBRA1 activation is to be used as a drug strategy. Since AMBRA1 is strongly expressed in mature neurons (Karlsson et al., 2021) and the main regions of the brain affected in neurodegeneration (Di Bartolomeo et al., 2010), future work should focus on determining whether AMBRA1^TAT^ also causes the phenotypic changes we observed in SHSY5Y cells in a more physiologically relevant cell type. While the structure of the AMBRA1/DDB1 complex has recently been resolved (Liu et al., 2023), future work should also focus on determining the AMBRA1/DLC1 structure. This would allow the design of PPI compounds to disrupt this interaction and, ultimately, to interfere with molecular mechanisms promoting neurodegeneration, thus providing therapeutic benefit.

## MATERIALS AND METHODS

### Cell culture

HEK293T (Sigma, 1202200) and SHSY5Y cells (Sigma, 94030304-1VL) were purchased from ECACC and confirmed to be mycoplasma free. SHSY5Y cells were cultured in Dulbecco's modified Eagle's medium (DMEM, Gibco, 31966-021) whereas HEK293T cells were cultured in DMEM (Gibco, 11330032). Both were supplemented with 10% FBS (Gibco, 11580516) and 1% penicillin-streptomycin solution (P/S, Gibco, 11548876). Cells were cultured at 37°C with 5% CO_2_.

Mouse neural stem cells (mNSCs) were purchased from R&D systems (NSC002). The cells were grown in proliferative conditions in complete basal media: DMEM/F12+HEPES+Glutamine (Gibco, 11330032), 0.5× B27 supplement (Gibco, 1530536), 0.5× N2 supplement (Gibco, 17502048) 0.1 mM 2-Mercaptoethanol (Gibco, 3150010), 1× MEM NEAA (Gibco, 11140050), 7.5% BSA (Thermo Fisher Scientific, 15260037), 1.45 g/l D-(+)-Glucose (Sigma, G8644) and 1× P/S. The basal media was supplemented with 10 ng/ml EGF (Peprotech, 315-09), 10 ng/ml FGF (Peprotech, 100-188) and 1 mg/ml laminin (Sigma, L2020) to grow cells in proliferative conditions. To induce quiescence, plated cells were transferred into complete basal media supplemented with 0.05 ng/μl BMP4 (R&D systems, 5020-BP), 20 ng/ml FGF and 1 mg/ml laminin (Martynoga et al., 2013). To reactivate quiescent NSCs, cells were passaged with accutase and replated in complete basal media with supplement for proliferative conditions.

### Lentivirus generation and transduction

Mutant AMBRA1 lentiviral plasmids (Table S1) were generated by GeneArt Gene Synthesis (Thermo Fisher Scientific). HEK293T cells were transfected with lentiviral plasmids and the appropriate packaging plasmids using turbofectamine (Origene, TR30037) according to the manufacturer's instructions. After 24 h, the medium was changed to normal HEK293T media. After an additional 24 h, the medium was either filtered onto SHSY5Y receiver cells or 10 ml was added to 2.5 ml PEG-IT reagent (Abcam, ab1025238) and stored at 4°C overnight. This viral supernatant was then concentrated by centrifugation at 3200×***g*** for 30 min at 4°C before being resuspended in 100 µl virus suspension solution (Abcam, ab102538), aliquoted and stored at −70°C. For transduction, 20 µl of this concentrated virus was added to 660 µl normal media which was added to a well of a six-well plate of cells and left overnight before topping up the media with 2 ml the next morning.

### Western blot analysis

For autophagy flux experiments, cells were treated with 100 nM bafilomycin (Sigma, B1793) in normal growth media for 4 h at 37°C. Cells were washed with PBS (Gibco, 15326239), detached with TrypLE and lysed in RIPA buffer (Thermo, 10017003) supplemented with protease inhibitors (Sigma, 4693116001) on ice for 15 min with intermittent vortexing. Samples were then centrifuged at 14,000 rpm for 5 mins at 4°C before the pellet was discarded. Protein concentrations were determined with a Pierce™ BCA assay protein kit (Thermo Fisher Scientific, 23225). The required sample volume was made up to 100 μl using sample, dH2O with NuPAGE LDS sample buffer (4×; Invitrogen, NP0007) and NuPAGE sample reducing agent (10×; Invitrogen, NP0004). Cell extracts were separated by sodium dodecyl sulphate–polyacrylamide gel electrophoresis (Novex gels catalogue number NP0335, SDS–PAGE). The membranes were blocked in PBS blocking buffer (LI-COR, 927-70001) for 30 min at room temperature before being immunoblotted using primary antibodies at their optimal dilution (Table S2, LC3-II for autophagy flux experiments) in PBS blocking buffer for approximately 4 h at room temperature and then overnight at 4°C. The membranes were then washed with PBS and 0.1% Tween-20 (Sigma, P1379-100) before being incubated with secondary antibodies (Table S2) for 1 h at room temperature and washed again. Immunoreactive bands were imaged using Odyssey CLx (LI-COR Biosciences). The band intensity was semi-quantified by normalising with an endogenous control protein (GAPDH) using ImageStudio Lite (version 5.2.5 9; LI-COR Biosciences).

Autophagy flux was measured by quantifying the dynamic change in LC3-II expression following bafilomycin treatment. Bafilomycin causes a late-stage blockage in autophagy, inhibiting autophagosome lysosome fusion, which results in LC3-II accumulation. LC3-II expression, normalised to the endogenous control, was quantified and the difference between the bafilomycin treated and untreated samples created an autophagy flux score. This score was then used to compare basal autophagy flux between cells expressing various AMBRA1 mutants.

### Co-immunoprecipitation (CoIP)

SHSY5Y cells were pelleted and solublised at 4°C for 30 mins. Insoluble material was then removed by centrifugation at 1200 rpm for 5 mins. AMBRA1 antibody (Proteintech, 1376-1-AP) was then coupled to dynabeads according to the manufacturer's instructions (Thermo Fisher Scientific, 14311) before protein lysate was mixed with the beads. This was followed by multiple wash steps to collect the purified protein complex. The protein concentrations of the samples were then determined using a BCA assay and the samples were run on a western blot for the protein of interest as described above.

### Immunocytochemistry (ICC)

SHSY5Y cells were grown in 96-well CellCarrier Ultra plates (Perkin Elmer, 74004), coated with laminin (Sigma, L2020). Cells were then washed in PBS, fixed in 4% PFA (Alfa Aesar, 43368) in PBS for 15 mins, washed with PBS and blocked and permeablised for 1 h in PBS containing 10% donkey serum (DS, Sigma, S30-M) and 0.1% Triton-X-100 (Thermo, T/3751/08) in PBS. Cells were incubated overnight with primary antibodies (Table S2) in PBS containing 0.02% Triton X-100 and 2% DS at 4°C. After washing, secondary antibodies (Table S2) were applied for 1 h in PBS containing 0.02% Triton X-100 and 2% DS. Cells were stained with Hoescht 33342 (1:2000, Abcam, ab228551) for 10 min before the final wash steps. Plates were imaged on the INCell Analyzer 6500 HS with a 40× objective that captured eight widefield, single plane fields of view per well, and quantitative analysis was performed using Columbus (PerkinElmer).

### Caspase assays

SHSY5Y cells were plated on black clear-bottomed plates (Fisher, 10601442). After 24 h, cells were stained with NucRed™ Live 647 ReadyProbes™ Reagent (2 drops/ml, Sigma, R37106) and CellEvent™ Caspase-3/7 green ReadyProbes™ Reagent (2 drops/ml, Sigma, R37111) for 1 h. Staining was removed and cells were then treated with 0.1% DMSO (Sigma, D2650). The plate was imaged and analysed using the IncuCyte.

### mtKeima mitophagy assays

SHSY5Y cells were transduced with mtKeima virus as described above and left for at least a week before analysis. Cells were plated at 10,000 per well in PDL coated 96-well CellCarrier Ultra plates (Perkin Elmer, 16230551), left to settle for 24 h and then treated with 1uM CCCP for 24 h in 1% FBS containing media. Cell nuclei were stained with 1:1000 Hoescht 3342 for 10 min prior to imaging. Cells were imaged in 1% serum media (Gibco, 31966021) using the INCell Analyzer with a 40× water immersion objective. Randomization of imaging fields was performed through an automated function of the INCell software. In the 488 nm channel, which excites mtKeima in neutral environments, mtKeima shows a tubular network-like mitochondrial staining pattern which was labelled as ‘healthy mitochondria’. In the 561 nm channel, which excites mtKeima in acidic environments, the staining was more punctate and was labelled as ‘mitolysosomes’. Analysis was performed in the Columbus Image Data Storage and Analysis System (Perkin Elmer). Ratiometric analysis of red:green pixel intensity was performed to gain a more robust measure of mitolysosome punctae. The total area of the healthy mitochondria and mitolysosome punctae was quantified and a mitophagy index score calculated by using the ratio of mitolysosome: healthy mitochondrial area.

### qPCR

RNA was isolated using the RNeasy Micro Kit (Qiagen, 74004). cDNA was then reverse transcribed from 200 ng RNA using Superscript IV VILO MasterMix (Thermo Fisher Scientific, 11755250) on the ProFlexTM PCR System (Life Technologies). Triplicate samples containing the appropriate Taqman primers [Ambra1 (Mm00554370_m1) and Beclin1 (Mm01265461_m1)], Applied biosystems Taqman fast advanced mastermix (Thermo Fisher Scientific, 4444557) and 2 µl cDNA were analysed by qPCR using β-actin [Taqman, (Mm02619580_g1)] as a housekeeping gene and the following programme: 50°C 2 min, 95°C 20 s, 40 cycles (95°C 1 s, 60°C 20 s). QuantStudio7 was used to perform the qPCR and the 2^−DDCt^ method was used for analysis.

### Ki67 proliferation assay

mNSCs were plated onto PEI and laminin coated 96-well CellCarrier Ultra plates (Perkin Elmer, 74004). After 24 h, a media change was carried out to induce quiescence. Cells were transduced with lentivirus after a following 72 h. 48 h post-transduction, cells were fixed with 4% PFA for 15 min. The fixed mNSCs were washed twice with PBS before permeablisation with 0.1% saponin (Thermo Fisher Scientific, 47036-50G-F) in 2% BSA (Sigma, A7030) for 1 h at room temperature. Ki-67 primary antibody diluted 1:200 in 0.1% saponin solution was added overnight at 4°C. Wells were washed in PBS and 100 µl of secondary antibody (1:1000, Table S2) diluted in 0.1% saponin solution was added to each well for 1 h at room temperature. Wells were washed before adding Hoescht 33342 (1:2000) for 10 min at room temperature. Finally, cells were washed with PBS and incubated at 4°C for imaging on the InCell Analyzer, and quantitative analysis was performed using Columbus.

## Supplementary Material

10.1242/biolopen.060380_sup1 Supplementary information

## References

[BIO060380C1] Aman, Y., Schmauck-Medina, T., Hansen, M., Morimoto, R. I., Simon, A. K., Bjedov, I., Palikaras, K., Simonsen, A., Johansen, T., Tavernarakis, N. et al. (2021). Autophagy in healthy aging and disease. *Nat. Aging* 1, 634-650. 10.1038/s43587-021-00098-4 34901876 PMC8659158

[BIO060380C2] Antonioli, M., Albiero, F., Nazio, F., Vescovo, T., Perdomo, A. B., Corazzari, M., Marsella, C., Piselli, P., Gretzmeier, C., Dengjel, J. et al. (2014). AMBRA1 interplay with cullin E3 ubiquitin ligases regulates autophagy dynamics. *Dev. Cell* 31, 734-746. 10.1016/j.devcel.2014.11.013 25499913

[BIO060380C3] Chaikovsky, A. C., Li, C., Jeng, E. E., Loebell, S., Lee, M. C., Murray, C. W., Cheng, R., Deumeter, J., Swaney, D. L., Chen, S. H. et al. (2021). The AMBRA1 E3 ligase agaptor regulates the stability of cyclin D. *Nature* 592, 794-798. 10.1038/s41586-021-03474-7 33854239 PMC8246597

[BIO060380C4] Cianfanelli, V., Fuoco, C., Lorente, M., Salazar, M., Quongamatteo, F., Gherardini, P. F., De Zio, D., Nazio, F., Antionioli, M., D‘Orazio, N. et al. (2015). AMBRA1 links autophagy to cell proliferation and tumorigenesis by promoting c-Myc dephosphorylation and degradation. *Nat. Cell Biol.* 17, 20-30. 10.1038/ncb3072 25438055 PMC4976803

[BIO060380C5] Di Bartolomeo, S., Corazzari, M., Nazio, F., Oliverio, S., Lisi, G., Antionoli, M., Pagliarini, V., Matteoni, S., Fuoco, C., Giunta, L. et al. (2010). The dynamic interaction of AMBRA1 with the dynein motor complex regulates mammalian autophagy. *J. Cell Biol.* 191, 155-168. 10.1083/jcb.201002100 20921139 PMC2953445

[BIO060380C6] Di Leo, L., Bodemeyer, V., Bosisio, F. M., Claps, G., Carreta, M., Rizza, S., Faienza, F., Frias, A., Khan, S., Bordi, M. et al. (2021). Loss of Ambra1 promotes melanoma growth and invasion. *Nat. Commun.* 12, 2550. 10.1038/s41467-021-22772-2 33953176 PMC8100102

[BIO060380C7] Di Rita, A., D‘Acunzo, P., Simula, L., Campello, S., Strappazzon, F. and Cecconi, F. (2018a). AMBRA1-mediated mitophagy counteracts oxidative stress and apoptosis induced by neurotoxicity in humen neuroblastoma SH-SY5Y cells. *Front. Cell Neurosci.* 12, 92. 10.3389/fncel.2018.00092 29755319 PMC5932353

[BIO060380C8] Di Rita, A., Peschiaroli, A., Acunzo, P. D., Strobbe, D., Hu, Z., Griber, J., Nygaard, M., Lambrughi, M., Melino, G., Papaleo, E. et al. (2018b). HUWE1 E3 ligase promotes PINK1/PARKIN-independent mitophagy by regulating AMBRA1 activation via IKKalpha. *Nat. Commun.* 9, 3755. 10.1038/s41467-018-05722-3 30217973 PMC6138665

[BIO060380C9] Fimia, G. M., Stoykova, A., Romagnoli, A., Giunta, L., Di Bartolomeo, S., Nardacci, R., Corazzari, M., Fuoco, C., Ucar, A., Schwartz, P. et al. (2007). Ambra1 regulates autophagy and development of the nervous system. *Nature* 447, 1121-1125. 10.1038/nature05925 17589504

[BIO060380C10] Fimia, G. M., Di Bartolomeo, S., Piacentini, M. and Cecconi, F. (2011). Unleashing the Ambra1-Beclin 1 complex from dynein chains: Ulk1 sets Ambra1 free to induce autophagy. *Autophagy* 7, 115-117. 10.4161/auto.7.1.14071 21079415

[BIO060380C11] Fimia, G. M., Corazzari, M., Antonioli, M. and Piacentini, M. (2013). Ambra1 at the crossroad between autophagy and cell death. *Oncogene* 32, 3311-3318. 10.1038/onc.2012.455 23069654

[BIO060380C12] Gillotin, S., Sahni, V., Lepko, T., Hanspal, M. A., Swartz, J. E., Alexopoulou, Z. and Marshall, F. H. (2021). Targeting impaired adult hippocampal neurogenesis in ageing by leveraging intrinsic mechanisms regulating Neural Stem Cell activity. *Ageing Res. Rev.* 71, 101447. 10.1016/j.arr.2021.101447 34403830

[BIO060380C13] Karlsson, M., Zhang, C., Mear, L., Zhong, W., Digre, A., Katona, B., Sjostedt, E., Butler, L., Odeberg, J., Dusart, P. et al. (2021). A single-cell type transcriptomics map of human tissues. *Sci. Adv.* 7, eabh2169. 10.1126/sciadv.abh2169 34321199 PMC8318366

[BIO060380C14] Leeman, D. S., Hebestreit, K., Ruetz, T., Webb, A. E., McKay, A., Pollina, E. A., Dulken, B. W., Zhao, X., Yeo, R. W., Ho, T. T. et al. (2018). Lysosome activation clears aggregates and enhances quiescent neural stem cell activation during aging. *Science* 359, 1277.83. 10.1126/science.aag3048 29590078 PMC5915358

[BIO060380C15] Liu, M., Wang, Y., Teng, F., Mai, X., Wang, X., Su, M.-Y. and Stjepanovic, G. (2023). Structure of the DDB1-AMBRA1 E3 ligase receptor complex linked to cell cycle regulation. *Nat. Commun.* 14, 7631. 10.1038/s41467-023-43174-6 37993427 PMC10665379

[BIO060380C16] Martynoga, B., Mateo, J. L., Zhou, B., Andersen, J., Achimastou, A., Urban, N., Van Den Berg, D., Georgopoulou, D., Hadjur, S., Wittbrodt, J. et al. (2013). Epigenomic enhancer annotation reveals a key role for NFIX in neural stem cell quiescence. *Genes Dev.* 27, 1769-1786. 10.1101/gad.216804.113 23964093 PMC3759694

[BIO060380C17] Strappazzon, F., Nazio, F., Corrado, M., Cianfanelli, V., Romagnoli, A., Fimia, G. M., Campello, S., Nardacci, R., Piacentini, M., Campanella, M. et al. (2015). AMBRA1 is able to induce mitophagy via LC3 binding, regardless of PARKIN and p62/SQSTM1. *Cell Death Differ.* 22, 517. 10.1038/cdd.2014.190 25661525 PMC4326578

[BIO060380C18] Tang, D. Y., Ellis, R. A. and Lovat, P. E. (2016). Prognostic impact of autophagy biomarkers for cutaneous melanoma. *Front. Oncol.* 6, 236. 27882308 10.3389/fonc.2016.00236 PMC5101199

[BIO060380C19] Vazquez, P., Arroba, A. I., Cecconi, F., De La Rosa, E. J., Boya, P. and De Pablo, F. (2012). Atg5 and Ambra1 differentially modulate neurogenesis in neural stem cells. *Autophagy* 8, 187-199. 10.4161/auto.8.2.18535 22240590

[BIO060380C20] Wang, C., Haas, M., Yeo, S. K., Sebti, S., Fernandez, A. F., Zou, Z., Levine, B. and Gian, J. L. (2022). Enhanced autophagy in Becn1(F121A/F121A) knockin mice counteracts aging-related neural stem cell exhaustion and dysfunction. *Autophagy* 18, 409-422. 10.1080/15548627.2021.1936358 34101533 PMC8942493

[BIO060380C21] Xia, P., Wang, S., Huang, G., Du, Y., Zhu, P., Li, M. and Fan, Z. (2014). RNF2 is recruited by WASH to ubiquitinate AMBRA1 leading to downregulation of autophagy. *Cell Res.* 24, 943-958. 10.1038/cr.2014.85 24980959 PMC4123297

[BIO060380C22] Yazdankhah, M., Farioli-Vecchioli, S., Tonchev, A. B., Stoykova, A. and Cecconi, F. (2014). The autophagy regulators Ambra1 and Beclin 1 are required for adult neurogenesis in the brain subventricular zone. *Cell Death Dis.* 5, e1403. 10.1038/cddis.2014.358 25188513 PMC4540193

[BIO060380C23] Ye, J., Tong, Y., Lv, J., Peng, R., Chen, S., Kuang, L., Su, K., Zheng, Y., Zhang, T., Zhang, F. et al. (2020). Rare mutations in the autophagy-regulating gene AMBRA1 contribute to human neural tube defects. *Hum. Mutat.* 41, 1383-1393. 10.1002/humu.24028 32333458

