# Supplementary material for: Disrupting the interaction between AMBRA1 and DLC1 prevents apoptosis while enhancing autophagy and mitophagy
Source: Biol Open. 2024 Nov 26;13(12):bio060380. doi: 10.1242/bio.060380 (PMC11625884; doi:10.1242/bio.060380)
Supplement: Supplementary information [file biolopen-13-060380-s1.pdf]

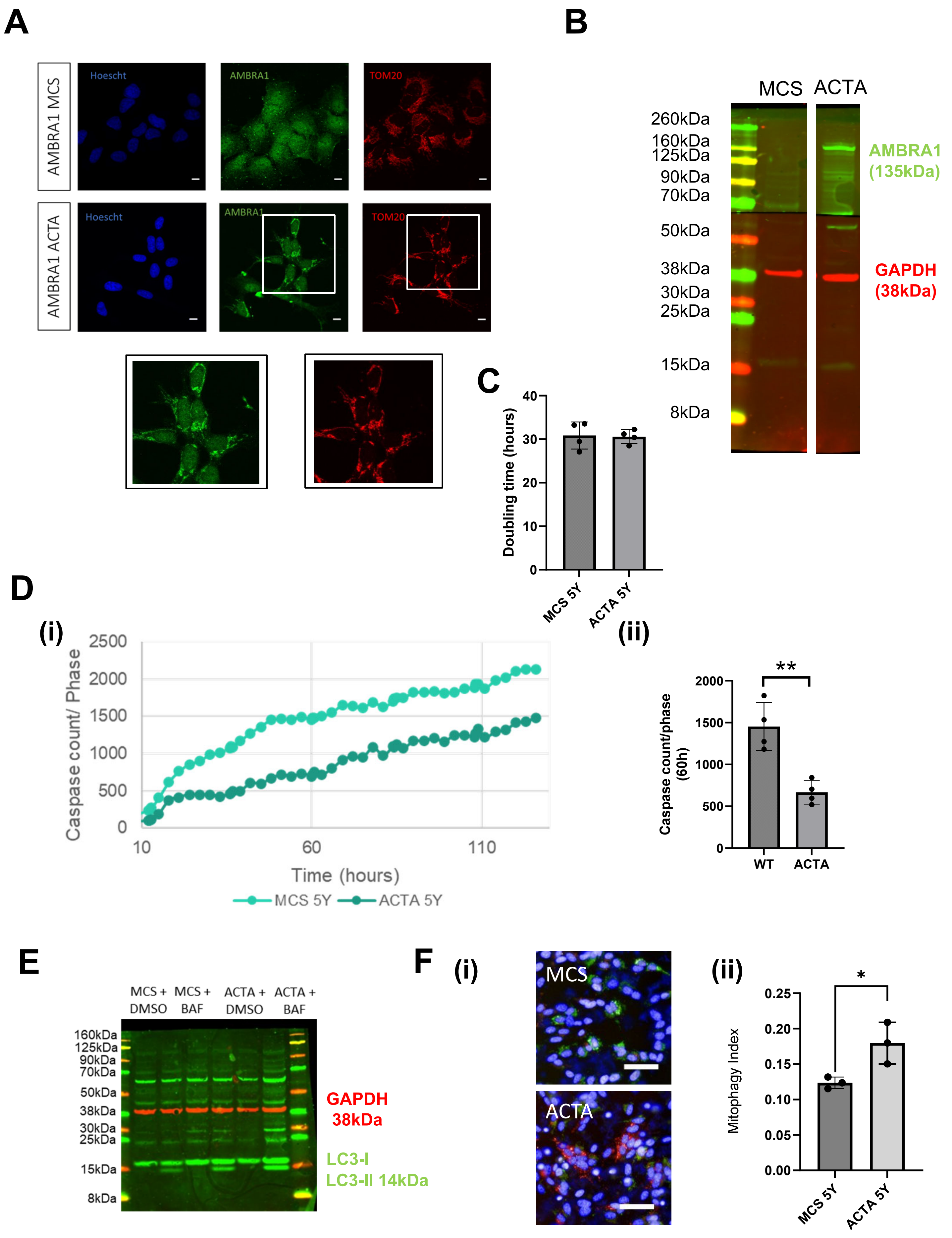

Fig. S1. AMBRA1^{ACTA} SHSY5Y validation and characterisation. (A) Immunostaining of Hoescht (blue), TOMM20 (green) and AMBRA1 (red) in AMBRA1^{ACTA} and MCS SHSY5Y cells. Scale bars =10um (B) Western blot of AMBRA1^{ACTA} and MCS SH-SY5Y cells to show AMBRA1 protein expression. (C) AMBRA1^{ACTA} and MCS SH-SY5Y doubling time measured in the Incucyte over a period of multiple days. (D) AMBRA1^{ACTA} and MCS SH-SY5Y cells stained with Caspase-3/7 Green and NucRed and imaged in the Incucyte for approximately 130 hours in growth media. Caspase levels were normalised to the phase area of each image. (ii) Sample mean ± SD. **p<0.01 (two tailed unpaired t test). (E) Western blot analysis of LC3-II expression in AMBRA1^{ACTA} and MCS SH-SY5Y cells treated with growth media and bafilomycin for 4 hours.. (F) Normalized quantification of the mitophagy index of AMBRA1^{ACTA} and MCS SH-SY5Y cells transduced with mtKeima virus and treated with 1uM CCCP**.** Scale bar = 50µm. Sample mean ± SD. Two two-tailed unpaired t-tests where *p<0.05. All data presented is representative of three experimental repeats. N=3.

Biology Open • Supplementary information

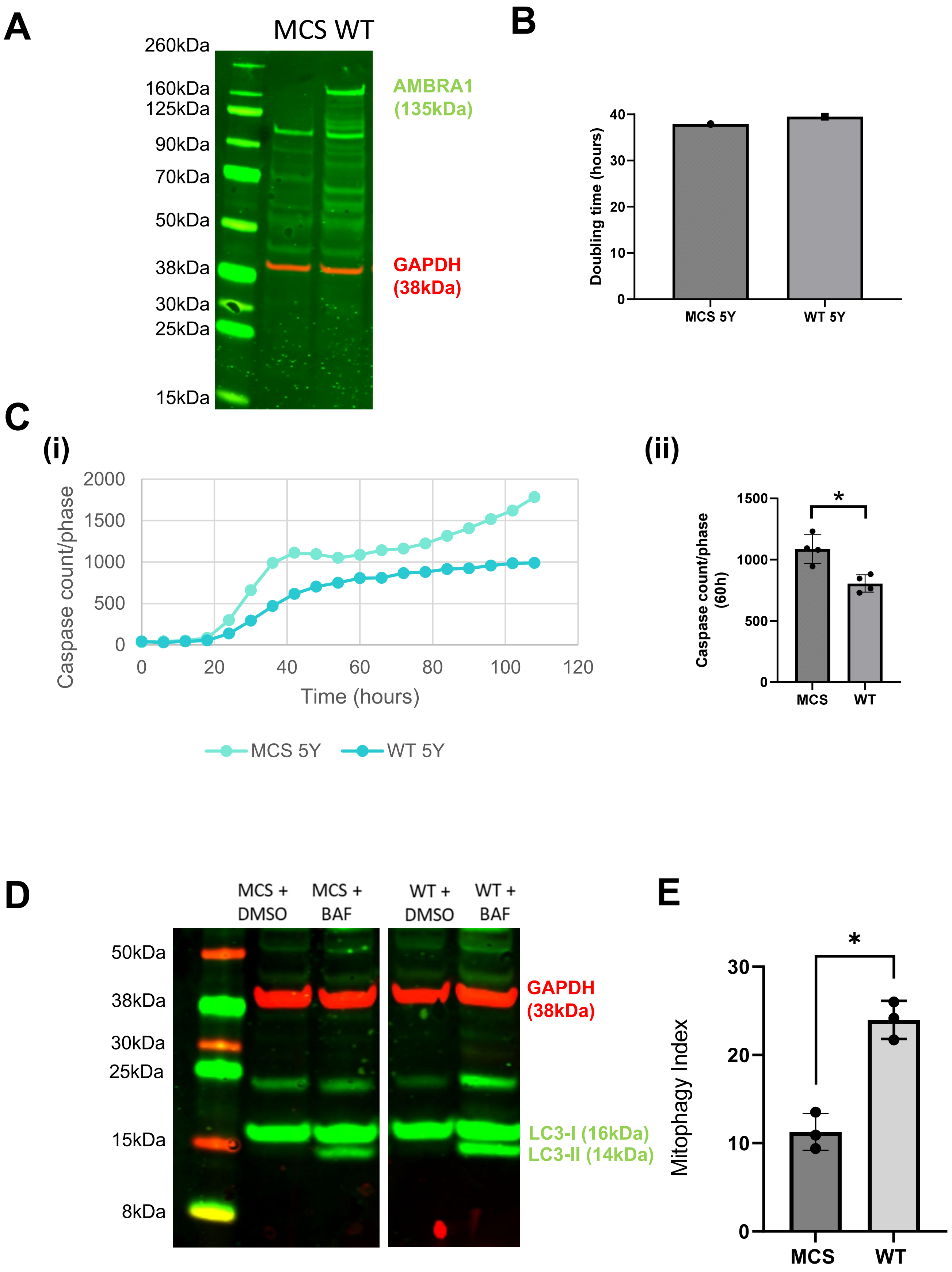

Fig. S2. AMBRA1^{WT} SHSY5Y validation and characterisation. (A) Western blot of AMBRA1^{WT} and MCS SH-SY5Y cells to show AMBRA1 protein expression. (B) AMBRA1^{WT} and MCS SH-SY5Y doubling time measured in the Incucyte over a period of multiple days. (C) AMBRA1^{WT} and MCS SH-SY5Y cells stained with Caspase-3/7 Green and NucRed and imaged in the Incucyte for approximately 130 hours in growth media. Caspase levels were normalised to the phase area of each image. (ii) Sample mean ± SD. *p<0.05 (two-tailed unpaired t-test). (D) Western blot analysis of LC3-II expression in AMBRA1^{WT} and MCS SH-SY5Y cells treated with growth media and bafilomycin for 4 hours. (E) Normalized quantification of the mitophagy index of AMBRA1^{ACTA} and MCS SH-SY5Y cells transduced with mtKeima virus and treated with 1uM CCCP**.** All data presented is representative of three experimental repeats. N=3. Sample mean ± SD. *p<0.05 (two-tailed unpaired t-test).

Biology Open • Supplementary information

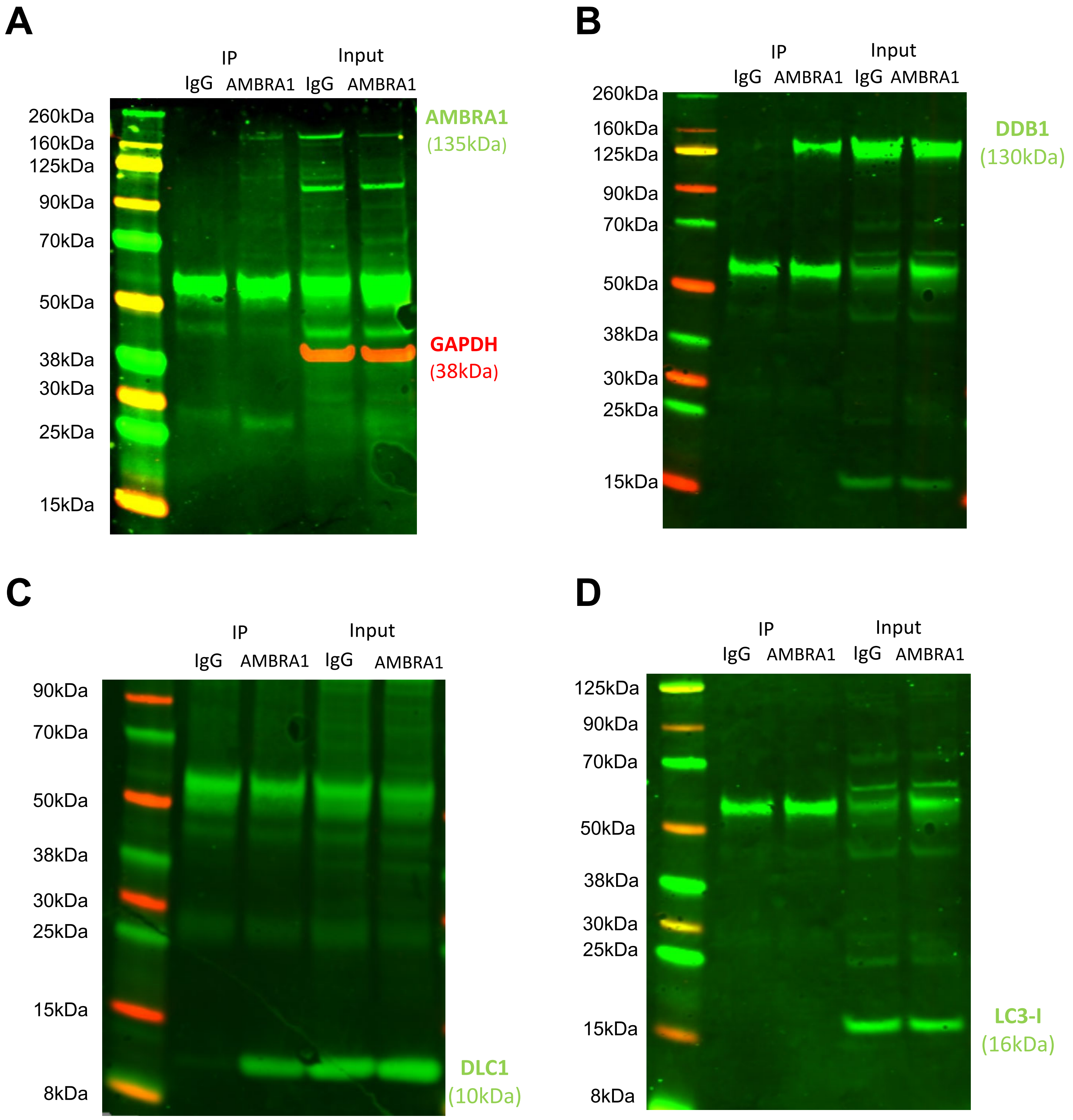

Fig. S3. CoIP controls. (A) A positive control showing that AMBRA1 has been immunoprecipitated when the AMBRA1 antibody but not IgG isotype control has been used and a negative control showing GAPDH in the input but not the IP fraction.(B) A negative control showing a band for DDB1 in the fraction bound to the AMBRA1 antibody (AMBRA1 IP) but not in the fraction bound to the rabbit isotype control antibody (IgG IP). (C) A negative control showing a band for DLC1 in the fraction bound to the AMBRA1 antibody (AMBRA1 IP) but not in the fraction bound to the rabbit isotype control antibody (IgG IP). (D) A negative control showing no band for LC3 in the fraction bound to the rabbit isotype control antibody (IgG IP).

Biology Open • Supplementary information

Table S1. AMBRA1 mutants.

Construct name	Publication	Sequences (mutated AA)	Expected consequence
TAT	(Di Bartolomeo et al., 2010)	SQGTATLALQ LQNAETATER	AMBRA1 translocates to the ER as it cannot interact with DLC1/2
ACTA	(Di Rita et al., 2018b)	TLILAMLAIGVFSLGAFIKIIQLRKNN	AMBRA1 translocates to mitochondria
WD40	(Antonioli et al., 2014)	IAAGCLDGEV RIWDLHGGSE	Cullin4 cannot degrade AMBRA1 as binding to DDB1 is impaired
S1014	(Di Rita et al., 2018b)	GDLVICRPEA LNDGVEYYWD QLNETVFTVH	AMBRA1 is active as it holds the same conformation as if it were phosphorylated by IKKα

Table S2. Antibodies

Antibody	Company, catalog number	Dilution
Anti-GAPDH Mouse Monoclonal Antibody	Abcam, ab8245	1:4,000
Recombinant Anti-DYNLL1/PIN Rabbit Monoclonal antibody	Abcam, ab51603	1:200
Recombinant Anti-DDB1 Rabbit Monoclonal Antibody	Abcam, ab109027	1:200
Recombinant Anti-LC3B Antibody	Abcam, ab192890	1:500
pBeclin-1 Rabbit Monoclonal Antibody	CST, 84966S	1:500
Anti-LC3B Rabbit Polyclonal Antibody	Novus Bio, NB100-2220	1:500
AMBRA1 Rabbit Polyclonal Antibody	Proteintech, 13762-1-AP	1:100
Total Beclin Rabbit PolyAB Antibody	Proteintech, 11306-1-AP	1:500
Ki-67 Rat Monoclonal Antibody	ThermoFisher, 14-5698-82	1:200
Donkey anti-rabbit mouse	Fisher, A21206	1:1,000
Donkey anti-mouse red	Fisher, A31571	1:1,000
IRDye® 800CW Donkey anti-Rabbit IgG	Licor, 15590485	1:4,000
IRDye® 680 RD Donkey anti-mouse IgG	Licor, 15550535	1:4,000
Rabbit IgG isotype control	Invitrogen, 31235	1:6 for CoIP

Biology Open • Supplementary information

Table S3. Autophagy data. LC3II/GAPDH.

**AMBRA1-TAT**	**Repeat 1**	**Repeat 2**	**Repeat 3**	**Average ± SEM**
**MCS**	2367	80	392	
**TAT**	3549	106	584	
**Fold change MCS**	1.00	1.00	1.00	
**Fold change TAT**	1.49	1.32	1.48	1.43 ± 0.06
**AMBRA1-ACTA**	**Repeat 1**	**Repeat 2**	**Repeat 3**	**Average ± SEM**
**MCS**	6883	3919	2273	
**ACTA**	8895	5239	2748	
**Fold change MCS**	1.00	1.00	1.00	
**Fold change ACTA**	1.29	1.33	1.20	1.27 ± 0.04
**AMBRA1-S1014**	**Repeat 1**	**Repeat 2**	**Repeat 3**	**Average ± SEM**
**MCS**	154	4815	5485	
**S1014**	209	7059	6202	
**Fold change MCS**	1.00	1.00	1.00	
**Fold change S1014**	1.35	1.46	1.13	1.31 ± 0.1
**AMBRA1-WD40**	**Repeat 1**	**Repeat 2**	**Repeat 3**	**Average ± SEM**
**MCS**	843	402	371	
**WD40**	484	321	183	
**Fold change MCS**	1.00	1.00	1.00	
**Fold change WD40**	0.57	0.80	0.49	0.62 ± 0.09

Biology Open • Supplementary information
